# “I am Your Mother and Your Father!” In Vitro Derived Gametes and the Ethics of Solo Reproduction

**DOI:** 10.1007/s10728-016-0321-7

**Published:** 2016-03-11

**Authors:** Daniela Cutas, Anna Smajdor

**Affiliations:** 1 Department of Historical, Philosophical and Religious Studies, Umeå University, Umeå, Sweden; 2 Department of Philosophy, Linguistics and Theory of Science, University of Gothenburg, Gothenburg, Sweden; 3 Norwich School of Medicine, University of East Anglia, Norwich, UK

**Keywords:** Solo reproduction, In vitro gametes, Reproductive cloning, Genetic account of reproduction, Single parenting, Motherhood, Fatherhood

## Abstract

In this paper, we will discuss the prospect of human reproduction achieved with gametes originating from only one person. According to statements by a minority of scientists working on the generation of gametes in vitro, it may become possible to create eggs from men’s non-reproductive cells and sperm from women’s. This would enable, at least in principle, the creation of an embryo from cells obtained from only one individual: ‘solo reproduction’. We will consider what might motivate people to reproduce in this way, and the implications that solo reproduction might have for ethics and policy. We suggest that such an innovation is unlikely to revolutionise reproduction and parenting. Indeed, in some respects it is less revolutionary than in vitro fertilisation as a whole. Furthermore, we show that solo reproduction with in vitro created gametes is not necessarily any more ethically problematic than gamete donation—and probably less so. Where appropriate, we draw parallels with the debate surrounding reproductive cloning. We note that solo reproduction may serve to perpetuate reductive geneticised accounts of reproduction, and that this may indeed be ethically questionable. However, in this it is not unique among other technologies of assisted reproduction, many of which focus on genetic transmission. It is for this reason that a ban on solo reproduction might be inconsistent with continuing to permit other kinds of reproduction that also bear the potential to strengthen attachment to a geneticised account of reproduction. Our claim is that there are at least as good reasons to pursue research towards enabling solo reproduction, and eventually to introduce solo reproduction as an option for fertility treatment, as there are to do so for other infertility related purposes.

## Introduction

Increasing use of assisted reproductive technologies (ARTs) over recent decades has brought many challenges to the practice, ethics and policy of human reproduction and parenting. ARTs have facilitated the separation of genetic and gestational motherhood; they have allowed people with very limited fertility to reproduce; they have increased the number of cases in which parents and children are not genetically related to each other. A few things however have stayed the same. So far, every child ever created has had two chromosomal parents—one of each sex.^1^

Success in current research in reproductive genetics however might change this. A few years ago, mice were conceived using genetic material from two males [19], and some scientists claim that both the generation of viable eggs from males and of sperm from females are feasible in human reproduction in the future [19, 39]. Hendriks et al. [38] describe a number of pathways towards the development of in vitro derived gametes in a systematic review published in 2015. They identify 9 biologically plausible routes that could lead to the development of artificial oocytes in males and 9 biologically plausible routes that could lead to the development of artificial sperm in females. According to Hendriks et al., clinical application is the expected outcome of this research. However, they note that the state of knowledge concerning functionality and safety of human in vitro derived gametes is still preliminary. Currently, research towards obtaining human gametes in vitro from other types of cells is ongoing in several labs around the world [10].

Some researchers have suggested that “self-fertilisation” is also possible in the future [16]: this could be achieved by using a gamete that an animal naturally has and an in vitro created complementary gamete, obtained from the cells of that same animal. If this is possible, then using the procedure in humans might also be possible. Such a prospect has been met with controversy and has been termed “the ultimate incest” [12]: a catchy phrase much cited in the British media in 2008. The prospective use in human reproduction of in vitro gametes, and in particular the derivation of eggs from male cells and of sperm from female cells, is looked upon with scepticism in the scientific community. In 2008, the Hinxton Group (a group of scientists and other experts, constituted at the initiative of the Stem Cell Policy and Ethics Program and the Johns Hopkins Berman Institute of Bioethics), issued a statement according to which

- It is likely to be very difficult to derive eggs that could be used for reproduction from XY (chromosomally male) cells.
- There are biological and technical reasons that will make it even more difficult, or even impossible, to derive sperm that could be used for reproduction from XX (chromosomally female) cells [40].

For the purposes of this paper, we will assume that functional human gametes, both eggs and sperm, *can* be obtained from somatic cells taken from either females or males, and they *can* be used in reproduction with gametes obtained from the same person. It would not be the first time scientific progress is made against expert predictions. Should this be the case, it could be of significant interest to people who currently view their reproductive options as being limited or non-existent. The ability to create eggs from somatic cells from males, and sperm from females, could offer same-sex couples the possibility to become (genetic) parents together [21]. Another possible use is that of creating gametes from one person’s cells and using them in reproduction with her own gametes. The use in reproduction of eggs from cells from men and sperm from women would create new legal and social challenges, and solo reproduction would be still more challenging. Perhaps the first question to be asked here is: why would anyone want to do this?

## Why Might Someone Want to Undertake Solo Reproduction?

When considering the possibility of solo reproduction one could ask whether there is a *need* for it. Since no-one is able to reproduce naturally without a partner, there is no obvious place for solo reproduction in medicine. Yet, as we have argued elsewhere [65], it is frequently the inability to fulfil one’s *reproductive aspirations* that drives people to the fertility clinic, rather than any specific medical disorder. The fulfilment of these aspirations depends partly on one’s circumstances. A woman may seek in vitro fertilisation (IVF) and intra-cytoplasmic sperm injection (ICSI) because of her husband’s incapacity to reproduce, even though she could reproduce unaided with a different partner. For this reason, the question to ask is not so much ‘who needs’ this technique, but ‘who might want it’?

Reproductive aspirations often have a genetic component: people wish to have a child genetically related to them and to their partner if they have one. But this is not always possible. For someone who does not specifically wish to have a child genetically linked to any other individual apart from herself, the possibility of solo reproduction may be highly appealing. One group of potential users could be couples in which one of the partners is unable or does not wish to reproduce or is at risk of transmitting a serious genetic condition to their offspring. Single individuals might likewise see merits in using only their own genetic material. In both cases, people might prefer to avoid having to deal with the legal and social ramifications of gamete donation and with whatever other problems might come via someone else’s DNA. Given the current significance with which genetic connections are invested in our societies—which includes the perception of genetic progenitors as a child’s *real* parents [31, 46]—having a child with the help of gamete donation is a challenge for life. Whether or not the parents are open with their children about their conception, whether or not the donors are known, the donation is rarely a one-off event that families can simply put behind them: it can affect the relationship between the parents, the parents’ relationship with their child, with their extended families, and with society in general [53]. Some of the reasons in favour of solo reproduction via in vitro created gametes may coincide with some of the reasons in favour of reproductive cloning: in the words of Lee M Silver writing in the context of reproductive cloning, “why should I put unknown, unneeded, potentially disease-causing genes into my child when I don’t have to?” [64] Reproducing with oneself would keep things very much in the family, so to speak.

Research indicates that some single women choose to have children while they still can, but hope eventually to find a (parenting) partner [30]. These women may have an extra reason to prefer to not complicate their family life by introducing an external thread of genetic parenting in their family if they can avoid it. In this way, even though their partner—if and when they find one—will not be the child’s genetic parent, at least no other party outside the couple will, either. There is no man out there who the child [or her parent(s)] might one day come to refer to as the *real* father. Single men might consider solo reproduction for similar reasons—although for the time being, in their case a woman’s contribution would still be required to carry the pregnancy.

Finally, donor gametes (especially eggs) are not always easy to come by and some people might have moral objections to using other people as sources of gametes. For example, they may think that that amounts to exploitation or instrumentalisation of another person. They might also think that their and their children’s lives will be easier without these additional connections and related difficulties, which may include rejection from these other parties if the child reaches out to them. With solo reproduction, there is no external narrative—no ‘other’ whose motives and aspirations might form a focus of interest either for the child or for the adults who are bringing up that child, so long as her genetic parent is one of them.

## Solo Reproduction and Reproductive Autonomy

Reproductive autonomy is often regarded as being of such importance that it should override moral objections to other people’s reproductive choices. One reason for claiming that reproductive decisions merit special respect is that they represent a particularly valuable area of human freedom [23]. In many cases, the arguments presented for prioritising reproductive decisions and reproductive freedoms focus specifically on the biological nature of reproduction [37]. One of the most cited accounts of reproductive autonomy, John Robertson’s, is rooted in biology and genes. According to Robertson, there is a ‘basic biologic […] drive to have a biologically related family’ [57]. Similar ideas can be found in policy documents. According to The Warnock Report, people experience ‘…a powerful urge to perpetuate their genes through a new generation. This desire cannot be assuaged by adoption’ [71].

But exactly what is encompassed by this principle is not entirely clear. Different aspects of biological and genetic relationships may be valued differently by prospective reproducers [3]. The woman who longs to gestate her child may do so in the near future through a womb transplant—and indeed the first live births by women having carried their baby in transplanted uteri have already taken place [8, 68]. The person who longs to become a genetic parent but who has no partner or whose partner is infertile may wish to reproduce with in vitro created gametes. ARTs have increased the scope for people to choose parenthood independently of the usual social and biological constraints. Possibilities that might not even be perceived as ‘reproductive’ by many or even most of us are not obviously outwith the bounds of others’ reproductive aspirations: the question depends on the contents of those aspirations, and these are not the same for each person.

John Harris argues that the principle of respect for reproductive autonomy could encompass reproductive cloning [33], and that the only grounds for preventing this would be the risk of harm to the resulting child [34, 41]. Although Harris does not fully articulate his reasons for categorising cloning as reproduction it seems reasonable to infer that it is primarily because reproductive cloning, like ‘natural’ reproduction, involves the transmission of genes from adult to child, with the intention to then rear that child. The WHO took a diametrically opposing stance, describing reproductive cloning as *replication* rather than reproduction and claiming that because it was not *really* reproduction it should be forbidden [71]. Human reproduction, it has been argued, is essentially collaborative and sexual, whilst cloning is not sexual and can be non-collaborative (when genetic material from only one person is used), and thus is more akin to manufacture than reproduction [52]. Furthermore, the *embryo* obtained through reproductive cloning is and is not an embryo, depending on how we define “embryo”: on the one hand, it is not because it does not arise from conception (the union of gametes); on the other hand it is, because it has the potential to develop into a human being [49]. Likewise, it could be argued that solo reproduction with in vitro gametes is not a form of reproduction and thus is not protected by reproductive autonomy. Indeed, solo reproduction would not precisely match *what we mean now* by reproduction. However neither did IVF or embryo transfer or other forms of altering the natural process of human reproduction. It is difficult to see why instead of regarding the established content of a definition as strictly unchangeable, we cannot look for the broader reasons why we would call something reproduction: for example, because it is a process through which someone becomes a (genetic) parent.

Many or most people who seek ARTs are specifically interested in the genetic aspect of reproduction. If genetic transmission is taken to be an essential component of reproduction for many fertility patients, it would appear that solo reproduction with in vitro created gametes could be classified straightforwardly as an additional assisted reproductive treatment. Several reasons could be advanced to support the statement that solo reproduction *is* reproduction and ought therefore to fall within the scope of endeavours protected by reproductive autonomy: it involves the transmission of genes from adult to offspring; it is the result of the fertilisation of an egg with a sperm; the embryo is gestated and the child born in the normal way. As long as genetic transmission remains the focus of many people’s reproductive aspirations, it is evident that solo reproduction with in vitro created gametes will be viewed as a desirable treatment by at least some people.

If an argument is to be made that solo reproduction should be allowed as a legitimate exercise of reproductive autonomy, are the risks of harm involved such that the argument would be compromised from the get-go?

## Risk

Establishing how much harm is required in order to justify the prohibition of a particular technique is challenging. We do not prevent people who carry genetic diseases that they risk passing on to their children (such as cystic fibrosis or Huntington’s disease) from reproducing. Most of us would not claim that it is unethical for them to do so—and few of those who *would* argue that it is, would take the additional step of claiming that they should be prevented from reproducing. Would the risks associated with recessive mutations in solo reproduction outweigh these risks? We must remember that we are talking about risks rather than certainties here. All pregnancies are risky; many parents risk passing on deleterious genes, or reproducing in socio-economic circumstances that are associated with a higher incidence of disease or difficulty to the resulting child [45]. Moreover, procedures such as ICSI and IVF are themselves known to be risky: a meta-analysis shows a 29 % increased risk of major malformation in offspring born as a result of these techniques, when compared with natural conceptions [56]. Yet these risks seem to be regarded as acceptable collateral damage both by clinicians and patients.

This is not to deny the biological complexities involved in reproducing with oneself. It is known that reproduction between close relatives increases the danger that offspring will inherit harmful mutations. These risks would be significantly magnified if an adult reproduced with him or herself, as it were. Solo reproduction would be akin to reproducing with one’s own identical twin. For many this might seem a sufficient reason to prohibit it. However, we suggest that the prospect should not be dismissed too hastily. Although it is well known that incestuous reproduction is risky, the exact degree of risk is hard to ascertain, and may be overstated [5]. A report published in 2011 suggests that the risk of congenital anomalies in first cousin marriages is 1.7–2.8 % higher than the background population risk [32]. Solo reproduction would probably be more risky than this—but the question of whether these risks are excessive, especially in comparison with already elevated risks of IVF and ICSI, cannot be a given. If, as John Harris suggests, risk is the sole ethical basis on which it may be justifiable to limit reproductive freedom, further work would need to be done to establish the degree of risk associated with solo reproduction in comparison with other, already accepted, reproductive techniques.

A particular complication in the context of weighing harm to offspring conceived using novel technologies is the so-called ‘non-identity problem’.^2^ In essence, if a child owed its existence and its identity to a technique which is thought to be risky—reproductive cloning, for example—it is hard to explain how the child has been *harmed* by the use of that technique [55]. If we had not used the technique, the child would never have existed at all. Because of this, Harris and many others believe that only if the suffering exceeds a certain threshold can we deem the child to have been harmed—and this would only be in cases where sickness or pain were so extreme that life itself is a burden to the child [35, 48]. Others have suggested that this threshold is illusory and that the reliance on ‘harm to offspring’ as the sole means of identifying unacceptable technologies is misguided [66]. The scope of this paper does not allow for a full analysis of these questions. It is sufficient for our purposes to note that there is no consensus as to how to identify and respond to excessive risk in reproduction.

Even if the risks involved in solo reproduction were known to be extreme, this would not necessarily imply that the technique must not be used at all. Genetic testing for autosomal recessive mutations could be carried out. Also, as we are looking at future possibilities, and at cases in which reproduction would take place in a clinical environment, there is nothing to prevent a very vigilant process of gamete and embryo screening, and prenatal diagnosis (PGD) could be used routinely in these cases. As pointed out by Palacios-Gonzales, Harris and Testa, “it is fair to expect that by the time that the prospect of IVG [in vitro gametes] for human reproduction is considered, we will have a grid of markers and assays to prospectively isolate the IVG that are most likely to result in viable healthy offspring. And it’s fair to note that this level of scrutiny is not even comparable to the one that accompanied the first-in-human application of IVF” [54]. Part of the appeal of in vitro created gametes is that they can be collected, multiplied and manipulated easily, and without risk or invasive procedures to the patient. So although the process is undeniably complex and costly, it would not be unrealistic to suppose that the risks could be brought within acceptable limits. (Testing and discarding large numbers of gametes and embryos might in its own right seem ethically questionable. However, the routine creation and disposal of surplus embryos is already an integral part of assisted conception in many countries where IVF is provided.)

It is important to acknowledge that opprobrium against incest is only partly rooted in concerns about genetic mutations, and indeed much predates knowledge about such risks. It also concerns family relationships and confusion of roles within the family and potential for abuse in unequal relationships (such as that between a parent and her offspring), parental responsibilities towards children (such as refraining from seducing one’s offspring) and the significance of trust in one’s upbringing, etc. Elective sterilisation or the possibility to successfully employ fertility treatment to avoid genetic mutations in offspring resulted from incest do not therefore entirely defuse objections against incestuous unions. Solo reproduction is incestuous in one sense: the parent reproduces with the most closely genetically-related person possible. However it exemplifies none of the problems outlined above. It *might* be criticised, like reproductive cloning has been before it [58], for displaying a form of narcissism and refusal of collaboration in reproduction: what is so good about your genes and so bad about mixing in anyone else’s that could make you want to reproduce with yourself? It may be that a person who would seek to be an only genetic parent may be embarking on such a journey with arrogant ideas about her own genetic structure. However, as we suggested above, this is not necessarily so: one might have reasons other than a love of one’s own genes to prefer to avoid donor gametes.

The prospect of creating a child who is *not* the genetic product of a 50–50 contribution from a male father and female mother might seem so abnormal or outwith natural processes as to be excessively dangerous. However, the exact 50–50 balance is not in fact a fixed feature of natural human reproduction, since a greater genetic contribution comes from the mother: offspring inherit the mother’s mitochondrial DNA as well as her chromosomes. Human reproduction is thus already genetically slightly unequal. In turn, this has made it possible recently to create offspring whose mitochondrial DNA is provided by a different woman from the chromosomal mother. Such individuals are the product of three adults’ genetic contributions. Even where there are only two genetic contributors, there can be variation, whereby offspring inherit more chromosomes from one parent than from the other. These anomalies can happen spontaneously in natural reproduction, and the imbalance can favour either the father or the mother [22, 67]. The main point here is that we already have ‘natural’ births that involve a higher proportion of chromosomes from one parent. It is not always 50–50, and the imbalance does not necessarily lead to drastic health problems in offspring. Whether there is a threshold beyond which significant harm would be caused, remains to be established.

Should the risks of solo reproduction for the health and wellbeing of the resulting children be higher than the alternatives for the prospective parent(s) (such as gamete donation), then solo reproduction with in vitro gametes might fall foul of what in bioethics has been called the Principle of Procreative Beneficence^3^ (PPB) [61]. According to this principle, when a choice is available, parents should choose the embryo that is most likely to have the best life. In its original formulation, the principle does not extend to a claim to outlaw the instances in which it is violated: so this objection would not suggest a need to outlaw the use of solo reproduction. Moreover, the principle has been formulated in the context of embryo selection where to exercise choice means simply to choose the ‘best’ embryo. If all the embryos are created using the solo parent’s cells, the PPB could be easily fulfilled by choosing the ‘best’ embryo from among those created. However, if we attempt to apply the PPB to the decision to embark on solo parenthood at all, it can easily be short circuited. The parent need only claim that they will either engage in solo reproduction, or forego reproduction altogether. Since the PPB does not demand that people forego reproduction, it would not therefore effectively rule out solo reproduction.

## Male Mothers, Female Fathers, and the Mother and Father in One

In recent years, the sexual dimorphism of genetic parenting is no longer necessarily reflected in legal ascription of parenthood: in some countries same-sex couples share legal parenting of their children. Moreover, parents can undergo gender reassignment procedures and thereby fathers may become mothers and vice versa. There have been cases in which female to male transgender people have given birth after their gender reassignment was recognised legally: thus becoming *birth fathers* (or *birth male mothers*). Such are the famous case of Thomas Beatie, the American man who gave birth to his and his wife’s three children, and more recent cases such as those reported in Germany and Israel [4, 42, 43]. Though some may disagree as to whether these protagonists are ‘really’ men,^4^ legally they are men who gave birth to children. Furthermore, Beatie refers to himself as his children’s father. Thus, there already *are* (genetic and gestational) ‘mothers’ who are their children’s fathers—if we equate male parent with father.^5^

A large and growing body of research indicates that what matters most for children is family functioning (the quality of relationships in a family) rather than family structure (the number, sex, or sexual orientation of the parents, whether or not parents and children are related genetically to each other) [6, 9, 26–28, 36, 44, 63]. More specifically regarding the gender of the parents, results have been slightly better when the parents were a lesbian couple [25], and also in the case of adoptive gay father families [29], than in the case of families with two parents of different gender. According to a research literature review by Biblarz and Stacey, “parenting skills are not dichotomous or exclusive”, and the gender of parents “has minor significance for children’s psychological adjustments and social success” [6]. According to a recent literature study, the “no difference” outcome for children from being raised by same-sex parents has reached scientific consensus [1]. These results give us reason to expect that the innovations that solo reproduction would bring in terms of parental gender are unlikely to have a devastating impact on the children.

One might object to the uniquely unusual situation that one’s genetic mother would *also* be one’s genetic father. How will children feel about this? Concerns such as these regarding the impact of unusual conception methods on children were brought to the surface at the beginning of IVF and other technologies that are in use today and which do not seem to cause the feared repercussions [18]. Many people are not comfortable thinking about the ways and circumstances in which they themselves were conceived, and this has not attracted attention as a good argument against natural reproduction. Moreover, concepts of motherhood and fatherhood have changed dramatically in the last decades [11], with fathers increasingly taking on caretaking tasks previously associated with motherhood—and indeed, being encouraged to become more motherly [20, 24]. Maternal and paternal roles are not fixed, and they vary across times, cultures, or personal circumstances. Having only one identifiable parent and only one that fulfils the parenting roles that in other families mothers and fathers fulfil together—an only parent who is *both one’s father and one’s mother*—is no novelty either.

It is difficult to substantiate a concern for the wellbeing of the children of solo reproduction in terms of parental gender(s). Solo reproduction may create new types of genetic connections, in a way similar to that in which IVF has allowed to split biological motherhood into two: the one that provided the egg and the one that carried the pregnancy to term and gave birth. Instead of multiplying a child’s connections to other individuals, solo reproduction would reduce them and locate genetic motherhood and fatherhood in one person.

## The Ultimate Single Parent

Solo reproduction can facilitate an extreme form of single parenthood. Single parenthood is regarded as a serious problem for society at large, for single parents themselves, and for their children. From a purely practical point of view, being brought up by more than one person is desirable [13], and indeed children might be better off with more than two committed parents [15]. The children of single parents tend to do less well at school, as well as suffering from a number of other social problems [2, 17, 60, 62, 69]. Furthermore, the idea that children need identifiable genetic parents is reinforced by the increasingly widespread requirement in Europe that sperm and egg donors should no longer be anonymous. Clearly, however, some of these concerns do not apply if there *is* no other parent or gamete donor. One’s origins would be *more* transparent if in vitro derived gametes were used for solo reproduction rather than donated gametes.

When people choose to become single parents through fertility treatment, they and their children do not experience the same problems as those from non-elective single parent families, and seem to achieve similar outcomes to offspring from well-functioning two-parent families [30, 47, 51]. Reproductive technologies, including prospective in vitro created gametes, allow single parenthood to be meticulously planned, thus avoiding the negative factors associated with unplanned single parenthood. Such factors may include relationship breakdown, death of a partner and other traumas which affect the remaining parent and children directly or indirectly. Where single parenthood is chosen there is no traumatic upheaval associated with the loss of a parent. Women will not be taken by surprise by their pregnancy, but will be able to plan ahead to ensure that adequate financial arrangements are in place. Single parenthood is not *necessarily* associated with harm to offspring. Again, it seems that in the case of in vitro derived gametes, many of these problems would either not arise, or would be significantly mitigated by the fact that parents would by necessity have had to consider, plan and budget for their reproductive projects.

In addition, it is important to note that solo reproduction does not entail single parenting. The genetic parent may share the parental role with her infertile partner or with someone else—similarly reproduction does not necessarily entail parenting and parenting does not necessarily presuppose having reproduced. Furthermore, discourses of reproduction and parenthood often seem to assume that children are raised in a kind of social vacuum, occupied only by their genetic or legal parents, but as Amy Mullin points out, this is simply false. It is often the case that many people contribute to the upbringing of children, even in the most nuclear of families. It is misguided, as well as unrealistic, to believe that a child’s parent(s) can or should provide *everything* that a child needs [50].

There are far fewer examples of single men than women using ARTs. However, it does occasionally happen, and there are reasons to believe that intentional single fatherhood is on the increase [7, 14]. For many people, motherhood and childhood are so intertwined that it may be hard to see how a man *could* raise a child alone. If the required in vitro derived gametes were available, the use of donor eggs would not be an issue. But if single men’s reproductive aspirations are to be fulfilled using in vitro derived gametes, this will, for the time being, require the use of surrogate mothers. This raises further ethical issues, which will also need to be considered.

We tend to regard single fathers as being admirable. Even more than with single mothers, there is often an assumption of some kind of tragedy or trauma that has led to the unfortunate father’s predicament. Again, however, in vitro derived gametes remove the element of tragedy or trauma and raise the question whether being brought up by one or more men is traumatic, or tragic, *independently* of whether one has experienced the loss of one’s mother. There is very little data on the welfare of children brought up by single fathers. Historically, children of men whose wives died would very rarely have been raised by their father alone. They would have been raised by female relatives or by the father’s new female partner. But gender expectations are changing, and it is no longer obvious that men are unable or unwilling to take care of a child without significant maternal input from women.

One important consideration in the kind of single parenthood that in vitro derived gametes could facilitate is the very specific way in which they enable *deliberate* choice of single reproduction. A parent who chooses to reproduce in such a way imposes on their child the unique situation of not having another genetic parent in the world, and implicitly no other genetic family thread to which she can relate. It is not that the other genetic parent is uninvolved in their upbringing or is unknown or has died: there is not, and there has never been one. The child might still acquire a second social or legal parent, but not a second genetic parent. Genetic relatedness, the capacity to recognize one’s traits in others and to learn one’s family history are valuable experiences: so much so that, according to the philosopher Velleman [70], intentionally alienating children from their biological relatives by creating them with donor gametes is immoral. Having knowledge of, and contact with, two branches of genetic relatives, can enrich one’s personal identity. Solo reproduction, while not estranging the child from genetic relatives, limits this possibility. An adopted child or a child born via anonymous donor gametes or who is estranged from her biological relatives may hope to find them, whereas nothing may be done in this regard for the child of solo reproduction.

In a way, the situation of this one-parent–child is similar to that of the cloned child, who is also the biological child of only one person. Inasmuch as cloning (almost) replicates a genetic make-up, the cloned child would be genetically the (almost) identical twin of her parent. Thus, it could be said that the clone’s *real* genetic parent is not the person whose genome has been replicated, but that person’s own parents. The child of solo reproduction however does not share an identical genome with her progenitor. Nevertheless, like the cloned child, she has as many genetic relatives as her parent. Not more, but also not fewer.

Another concern that may apply to solo reproduction via in vitro derived gametes as well as reproductive cloning is that it can be an expression of, and reinforce, a reductive proprietarian account of parent–child relationships: my genetic child is *more* mine than other children that I might raise; the child cloned from me or conceived from *my* cells *only*, is *even more mine* than one whose genetic parentage I share with someone else. Furthermore, prospective parents via solo reproduction may be acting out of a set of socially encouraged beliefs that the genetic connection between parents and children constitutes a necessary, or even the fundamental, ingredient of parenting. Yet fertility treatments *are* currently being used to enable people to become parents genetically. The implications of such an objection would need to reach all these other cases as well. It would be inconsistent to continue to support some people in their quest to have the genetic component in their reproductive endeavours, and deny it for others. The holding of a geneticised account of parent–child relationships is not intrinsic to solo reproduction, or to the preference to reproduce genetically, in general: as we have seen above, one may have a variety of reasons to prefer to reproduce in this way, many of which do not rely on prospective parents acting out of such a belief. Moreover, a charge *against* solo reproduction for representing “the ultimate incest” can itself be an example of such a geneticised account of reproduction and parenting.

## Conclusion

If one accepts the principle of reproductive autonomy, there are strong reasons for regarding solo reproduction as an endeavour to be protected as much as other forms of reproduction. It may be that solo reproduction with in vitro created gametes will never become feasible in humans, or that the risks will outweigh the potential benefits. However, as yet, the degree of risk is uncertain, and the hierarchy of reproductive risks is poorly articulated. There is clearly more work to be done in this quarter.

It is probably, all things considered, better for children to have more than one committed parent. This may maximise the chances of the child receiving more care, more resources, higher likelihood of parental survival, etc. However, single parenting need not trigger bad outcomes for children: especially when it does not come about due to tragic causes such as the death of a parent, and when the socio-economic circumstances in which it unfolds are good. Furthermore, single reproduction does not even entail single parenting—unless one adopts a very narrow genetic account of what parenting means. It might also be the case that it is better for children to have more than one *genetic* parent specifically. Having more than one genetic parent, and thus more than one set of genetic relatives, may allow more meaningful connections to more people.

Because solo reproduction reduces rather than expands the number and types of genetic connections that children will have, in some regards it simplifies rather than complicates things for the resulting child and her family. This may have disadvantages for the child (by not allowing her a possibly enriching experience) but also advantages (a stronger bond with the parent, the absence of genetic connections to donors who may not wish to be identified, contacted, or relate to the child).

## Footnotes

1 Technologies such as cytoplasmic transfer or mitochondrial transfer have often been reported in the media as creating “three parent children”. Whether the donor of mitochondria is thereby a parent is however controversial.

2 We thank an anonymous reviewer for raising this concern.

3 We thank an anonymous reviewer for raising this concern.

4 According to the judge presiding in Thomas Beatie’s divorce case, the divorce could not be granted because Beatie was a woman, and same-sex marriages were illegal in that state (Beatie was married to a woman). The reason why Beatie was not a man, in spite of being legally a man, was that men cannot bear children, and Beatie had [59].

5 In the UK women can be *female parents*: since 2008, this is the term used to denote the relation to the child of the same-sex partner of an IVF birth mother. The associations between woman parent/mother and male parent/father are therefore not necessarily reflected in the law.
